# Order within chaos: Harnessing *Plasmodium falciparum var* gene extreme polymorphism for malaria epidemiology

**DOI:** 10.1371/journal.pgen.1009344

**Published:** 2021-02-25

**Authors:** Marc-Antoine Guery, Antoine Claessens

**Affiliations:** 1 LPHI, Université de Montpellier, CNRS, Montpellier, France; 2 LPHI, MIVEGEC, Université de Montpellier, CNRS, Montpellier, France; Institut Pasteur, CNRS UMR 3525, FRANCE

## Genotyping methods

The characterization of *Plasmodium falciparum* genetic diversity is key to understand evolutionary pressure, measure the impact of elimination campaigns, monitor drug resistance, etc. A common genotyping method is to PCR amplify microsatellite markers or polymorphic genes, such as the *msp* family, the size of the resulting amplicon(s) being variable between different isolates [1]. Amplicon ultra-deep sequencing now allows multiplexing samples and are particularly useful to determine the proportion of each strain in infections with multiple genotypes [2]. Alternatively, genotyping a few dozen single nucleotide polymorphisms (SNPs) generates a molecular barcode specific to each isolate. Whole genome sequencing (WGS) is the most comprehensive approach, but the cost remains high for large-scale studies.

**Fig 1:**
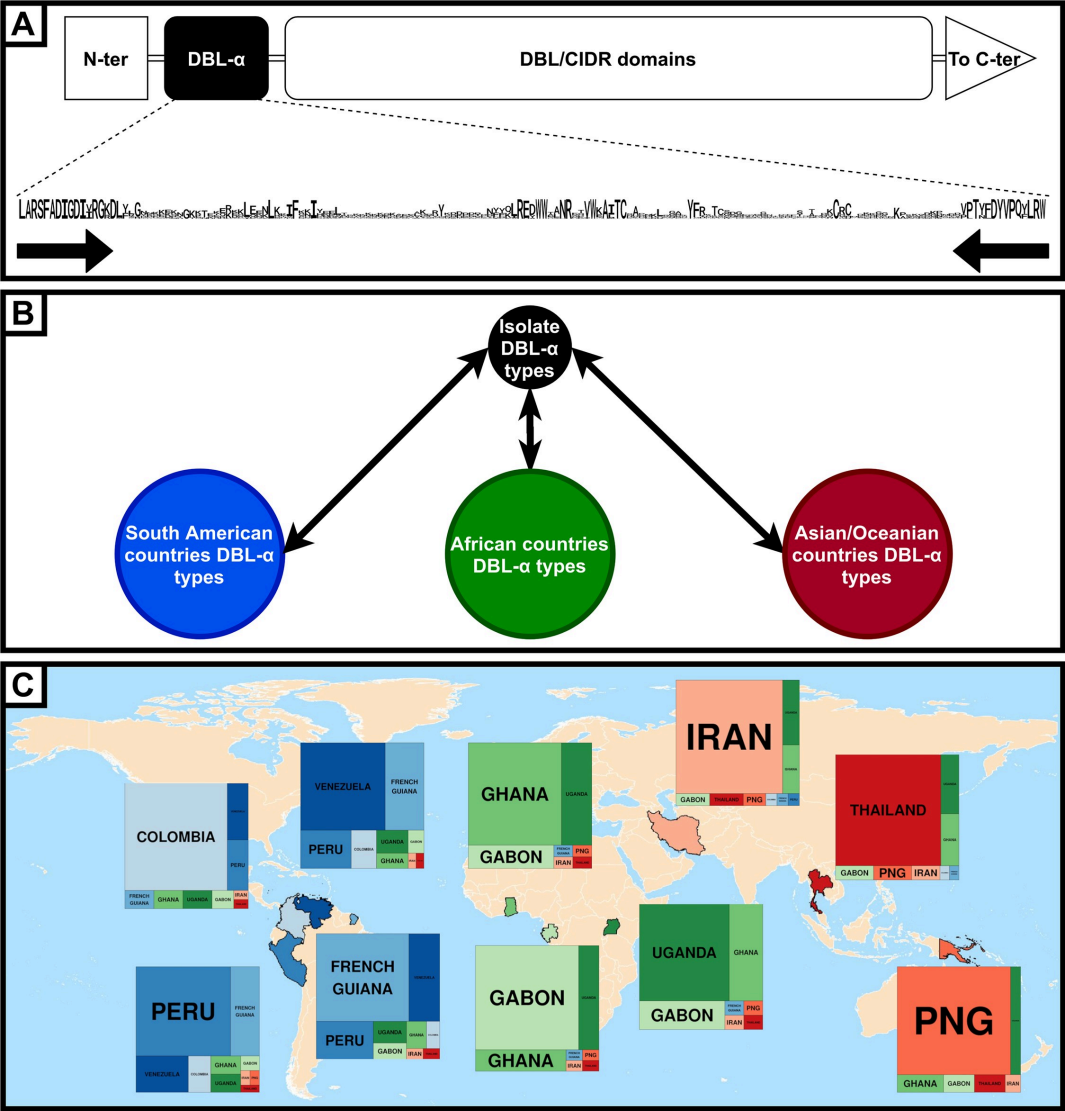
Relevance of using DBL*α* sequences for malaria epidemiology. (A) Schematic view of DBL*α* domain in PfEMP1, the protein encoded by *var* genes. The amino acid sequence of DBL*α* domain, with letter size proportional to the prevalence in the *P*. *falciparum* population, shows two conserved motifs on either side. Universal primers were designed to match the corresponding DNA sequence. (B) Each isolate’s geographical origin is estimated by comparing DBL*α* types found in the isolate with DBL*α* types found in a specific country. (C) World map with each country from South America (blue), Africa (green), and Asia or Oceania (red) highlighted. Rectangle areas are proportional to the weight of each country in the overall estimated geographical origin of isolates, based on DBL*α* matching proportions. For example, isolates from Uganda share a relatively high number of DBL*α* with Ghana and Gabon, while PNG is more isolated. Each country is preferentially self-matching. Data from Tonkin-Hill and colleagues [7]. Made with Natural Earth.

Over the last decade, the Day lab has pioneered a PCR approach based on *P*. *falciparum*’s most polymorphic gene family: the *var* genes [3]. The roughly 60 *var* genes have a similar organization consisting in a succession of Duffy binding-like (DBL) and cysteine-rich interdomain region (CIDR) domains with the near ubiquitous presence of DBL*α* subtype (Fig 1A) [4]. Through this approach, the DBL*α* domain is amplified using degenerate primers that match two short conserved motifs on either side of the domain. The approximately 450 bp-long sequence in between is extremely variable, making the total number of unique DBL*α* domains worldwide virtually infinite [5]. This cost-effective method is used as a surveillance tool across a variety of epidemiological settings. Unlike microsatellites that are presumably neutral markers, the DBL*α* typing offers the added advantage of characterizing the parasite’s most immunogenic surface protein family [6].

## DBL*α* sequences differentiate *Plasmodium falciparum* populations

To test the suitability of DBL*α* sequences for population genetics analysis, Tonkin-Hill and colleagues processed 32,682 sequences from 1,248 *P*. *falciparum* isolates collected in 10 different countries located in South America, Africa, Asia, and Oceania [7]. To account for the frequent recombination events between *var* genes, they developed a novel computationally intensive method known as jumping hidden Markov Model (JHMM) which is able to infer the posterior probability that each location in an isolate’s DBL*α* type amino acid sequence is most closely related to every other DBL*α* type [8,9]. They accumulated the probabilities between DBL*α* found in an isolate to provide an estimate of the expected proportion of relatedness between isolates. These proportions were then aggregated to provide estimates of an isolate’s DBL*α* repertoire that most closely matched each country, hence attributing a geographical origin to each isolate (Fig 1B). Country-specific clustering was observed, even in Africa where *P*. *falciparum* genetic diversity is particularly high [10], indicating that the majority of DBL*α* types are country specific. Excluding intracountry comparisons, countries located in the same continent show greater matching proportions of DBL*α* sequences, especially for Africa and South America. Regarding intercontinent comparisons, DBL*α* types coming from South America show greater relatedness to DBL*α* types found in Africa compared to Asia or Oceania (Fig 1C).

These results are in line with conclusions drawn from previous works, indicating that *P*. *falciparum* was introduced from Africa to South America during the slave trade [11]. Also, the JHMM proportions suggest that the *var* populations in Asia/Oceania more closely resemble African populations than those seen in South America, consistent with the expansion of *P*. *falciparum* out of Africa toward Asia [12]. Finally, the DBL*α* method is also able to distinguish between *Laverania* species.

## The exception to the rule: Conserved *var* genes

While most DBL*α* in the Tonkin-Hill dataset were detected only once, some appeared to be conserved at the global scale. The top 100 most prevalent DBL*α*, present in at least 50 isolates, were found to be distributed all over the countries sampled. This result was confirmed with a BLAST search against the NCBI and MalariaGEN-derived databases [13]. Although the in vitro recombination rate of group A *var* genes is lower than group B or C [9], the DBL*α*1 (specific to group A *var* genes) was proportionally represented in the Top 100. The overall mind-boggling genetic diversity of DBL*α* sequences make these specific “conserved” DBL*α* even more interesting.

What is driving this selection? Some DBL*α* could be in linkage disequilibrium with drug resistance alleles [14]. However, the authors showed that only 10% of conserved DBL*α* were associated with drug resistance markers, indicating that other selection forces are at play and remained to be discovered. Interestingly, they identified conserved DBL*α* on chromosome 6, in which long-range haplotypes have been reported in African *P*. *falciparum* genomes [15].

## Plasmodium diversity: A matter of scale

In summary, Tonkin-Hill and colleagues produced the first global scale DBL*α* types analysis. Their JHMM approach is promising when dealing with relatedness between sets of sequences, even though its computational cost remains to be lowered. Comparing DBL*α* sequences led to a clustering similar to what was observed using WGS in Africa, for a fraction of the cost. To further investigate the ability of DBL*α* comparisons to track *P*. *falciparum* populations, more samples are needed, especially from Ethiopia and the Democratic Republic of The Congo, where divergent parasites have recently been identified [10]. Follow-up studies should also include countries where malaria is highly seasonal, to measure the impact of transmission (or lack thereof) on parasite population genetic diversity.

To conclude, Tonkin-Hill and colleagues provide compelling evidence for further DBL*α*-based genotyping studies, not just for “varologists” but most epidemiological studies.
